# Supplementary material for: Enhanced RNA quality control maintains long-term regenerative ability in planarians
Source: Development. 2025 Oct 16;152(20):dev204762. doi: 10.1242/dev.204762 (PMC12579931; doi:10.1242/dev.204762)
Supplement: Supplementary information [file develop-152-204762-s1.pdf]

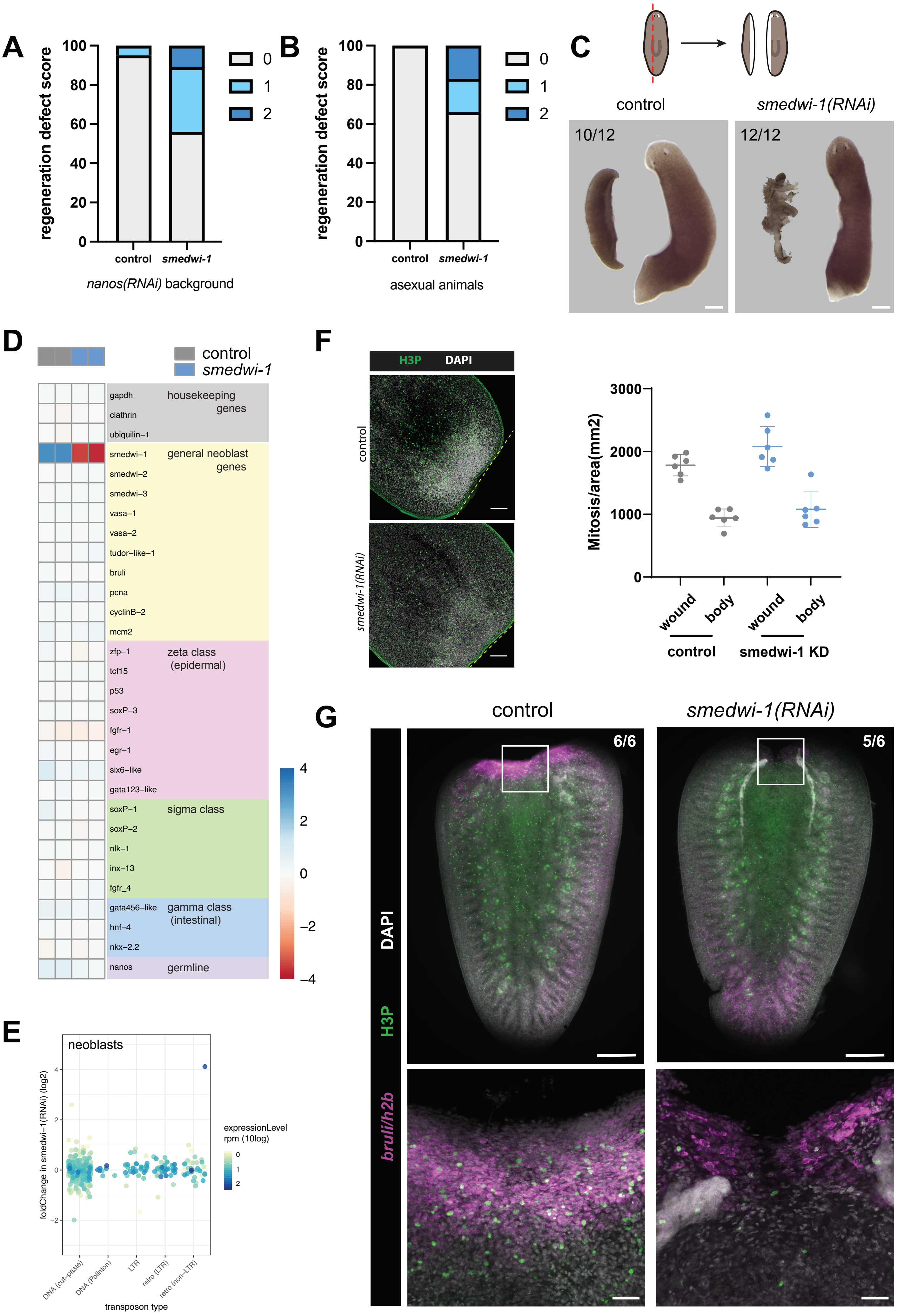

Development • Supplementary information

Fig. S1. (Ad Fig. 1)

A. Quantification of the frequency of observed wound healing phenotypes at d3 post amputation of sexual animals treated for 3 months with *nanos(RNAi)* to eliminate the germline, or with *nanos-smedwi-1(doubleRNAi)* treatment. Wound size was around 3mm. >20 animals per condition. Fisher's exact test, * $p<0.05$, **** $p<0.0001$.

B. Quantification of the frequency of observed wound healing phenotypes at d3 post amputation of asexual animals after 2 months of the *smedwi-1(RNAi)* treatment. Wound size was around 2mm. >20 animals per condition. Fisher's exact test, * $p<0.05$, **** $p<0.0001$.

C. Image of asexual control or *smedwi-1(RNAi)* animal at 24 hours after parasagittal section. Among control animals 10 out of 12 small lateral fragments survived and eventually regenerated. Among the *smedwi-1(RNAi)* animals all small lateral fragments lysed within 24 hours.

D. Heatmap of RNA expression levels of control genes and neoblast genes in control and *smedwi-1(RNAi)* neoblasts as determined by RNAseq analysis. Shown are log2 fold changes relative to the averaged expression.

E. Effect of long-term loss of SMEDWI-1 on transposon levels in isolated neoblasts. Transposons are classified by type and their baseline expression level is shown by coloring. No major changes in transposon levels are detected.

F. Immunofluorescence of phosphorylated histone 3 (H3P) in small tail pieces at day 3 after amputation (right), and quantification of the density of mitotic figures (left) in *smedwi-1(RNAi)* animals compared to controls. Scale bar, 300µm. Datapoints represent biological replicates (n=6).

G. (expanded from main Fig. 1f) Immunofluorescence of phosphorylated histone 3 (H3P) and FISH of *bruli* and *h2b* to mark neoblasts at day 3 after amputation of large posterior fragments. *smedwi-1(RNAi)* fragments have reduced accumulation of neoblasts at the wound site. Some background staining (out of focus) is present in the tail region of the animals. Clusters of H3P cells located along the flanks of the animals reflect the testes. White tracks at the top of the *smedwi-1(RNAi)* fragment are the sperm ducts. Scale bar, 1mm (top panels), or 50µm (bottom panels).

Development • Supplementary information

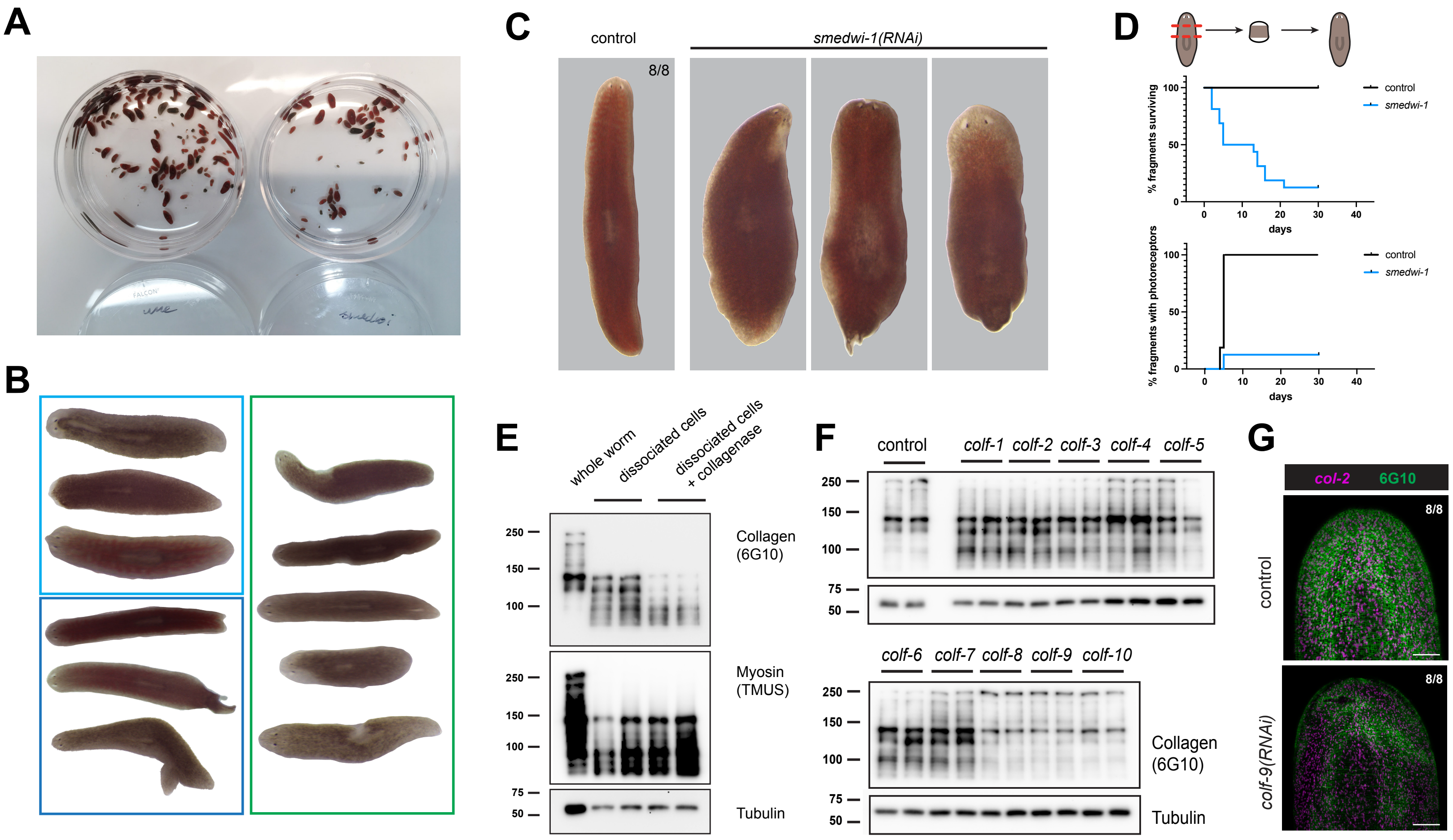

Fig. S2. (Ad Fig. 1)

In this study we focused on the wound regeneration phenotype that develops over time in *smedwi-1(RNAi)* animals. We did however observe several other signs of decreased health and increased fragility in these animals over time. We did not detect overt lethality among uninjured homeostatic *smedwi-1(RNAi)* animals when cultured in the presence of the antibiotic Gentamycin. However, in the *smedwi-1(RNAi)* asexual cultures the various non-regenerating fragments that were formed over the course of the animal culture eventually died, and fragments with large wound surfaces frequently lysed and dissolved (see for example **Fig. S1C**). In agreement with this, even in the absence of intentional amputations, colonies on *smedwi-1* dsRNA expanded notably less than colonies on control dsRNA.

Further, we found that homeostatic *smedwi-1(RNAi)* animals became more sickly than control RNAi animals over time, and showed increased incidence of morphological aberrations (found in around 15% of long-term *smedwi-1(RNAi)* animals whereas they are very rare (<1%) in controls. Further, in the absence of antibiotics, lysis of cut fragments and development of abnormalities was exaggerated in the *smedwi-1(RNAi)* animals, whereas no changes were observed in the controls, indicating that the *smedwi-1(RNAi)* animals were more fragile in the face of microbial challenges.

A. Illustration of the reduced expansion of *smedwi-1(RNAi)* asexual cultures compared to controls. Each culture was started as a cohort of 20 asexual worms and maintained on weekly feedings with control food or *smedwi-1* dsRNA for 6 months. The culture on control food expanded significantly more than the culture on *smedwi-1*dsRNA.

B. Illustration of the morphological aberrations developed in *smedwi-1(RNAi)* asexual cultures over time. Animals had been maintained on weekly feedings with *smedwi-1* dsRNA for 6 months. Animals with aberrations in head morphology (light blue box), tail morphology (dark blue box), or pigmentation defects (green box) were readily detected in the population whereas no such animals were found among controls.

C. Groups of eight 2-month control or *smedwi-1(RNAi)* asexual animals were maintained in planarian water without the antibiotic Gentamycin for 4 weeks. The control animals showed no defects, but out of the 8 *smedwi-1(RNAi)* animals, 2 shrunk and died, and 3 (shown) developed morphological defects.

Development • Supplementary information

D. Progression of regeneration of small tissue fragments (double wound site) from asexual animals in the absence of the antibiotic Gentamycin. Control tissue fragments all survived and regenerated heads with detectable eye spots in 5 days. Of the *smedwi-1(RNAi)* tissue fragments only 2 out of 16 regenerated a head and survived. The remaining *smedwi-1(RNAi)* fragments either lysed or aborted regeneration and shriveled away.

E. Treatment of macerated planarian cells with collagenase resulted in a reduction of the 6G10 signal on Western blot, suggesting that 6G10 recognizes an extracellular protein. Intracellular proteins Tubulin and Myosin were not affected by collagenase treatment.

F. Western blot of asexual animals treated with dsRNA against fibrillar collagens 1-10, labeled by the 6G10 antibody. Biological duplicates are shown. Knockdown of *colf-8*, *colf-9* and *colf-10* resulted in reduced signal on the blot.

G. Immunofluorescence of animals treated with dsRNA against *colf-9* showed no change in the number of muscle cells as marked by *collagen-2* mRNA, but showed reduced staining by antibody 6G10. Scale bar, 100µm.

While the exact epitope for the 6G10 antibody remains unknown, based on our data we propose that the target is a collagen.

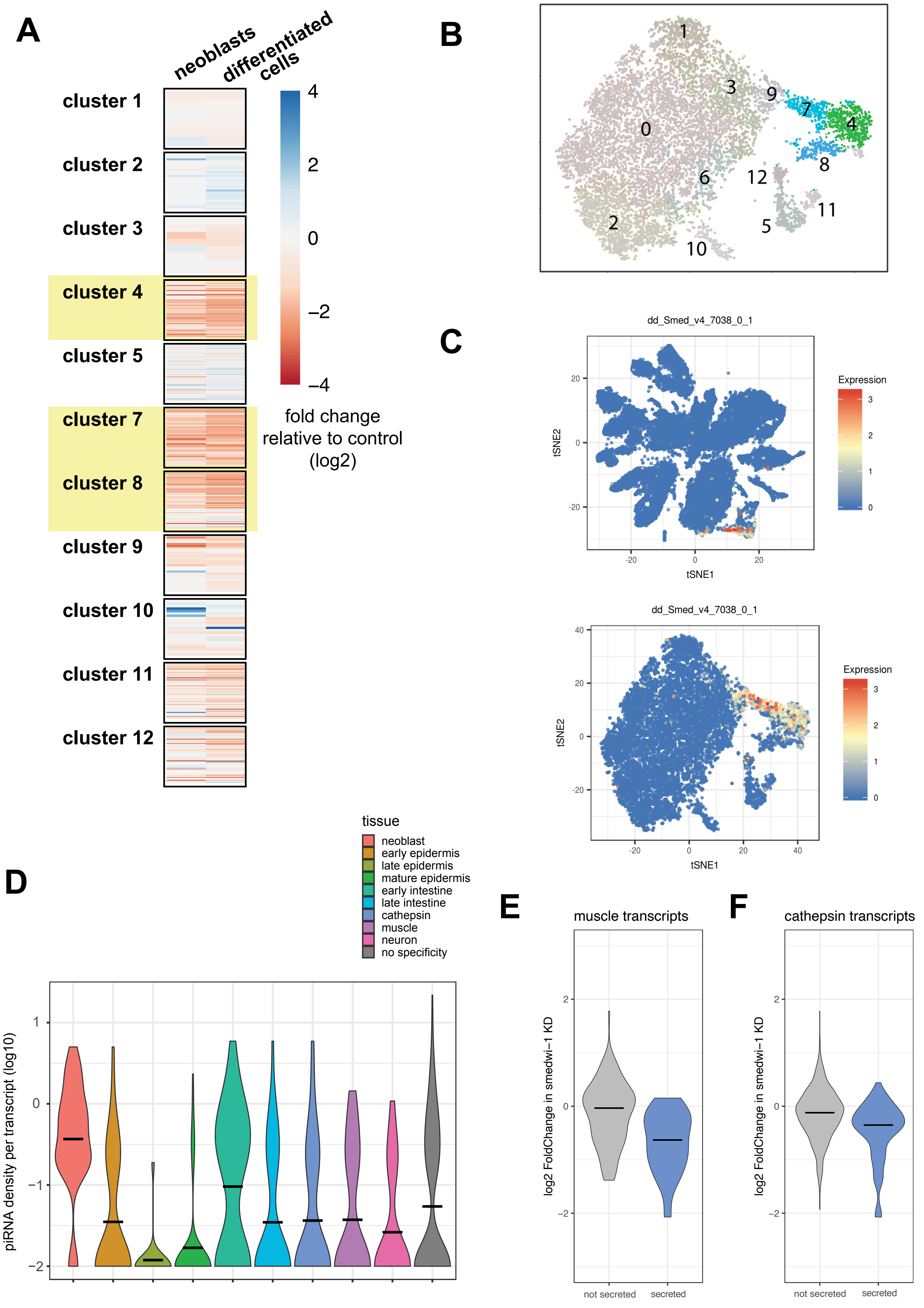

Development • Supplementary information

Fig. S3. (Ad Fig. 3)

A. (expanded version of main Fig. 3b) Analysis of genes characteristic for subclusters of epidermal cells as determined by single cell sequencing (Fincher et al., 2018), shows that transcripts altered in *smedwi-1(RNAi)* animals are largely confined to a few clusters, which mark a late epidermal precursor stage, known as "category 3 cells".

B. UMAP representation of the epidermal subclusters (Fincher *et al.*, 2018) showing that affected transcripts are found in a specific subset of epidermal cells.

C. UMAP representation of total cells (top, (Fincher *et al.*, 2018)) and epidermal subclusters (bottom, (Fincher *et al.*, 2018)) marking the cells that show expression of the transcription factor EGR-5. EGR-5 expression is restricted to a specific group of cells, known as the "category 3 cells".

D. Violin plot showing the density of piRNAs per transcript in each cell type of wildtype animals. Horizontal bars indicate the median number of piRNAs per transcript for each tissue. Transcripts that are specific to late epidermal precursors tend to have low levels of matching piRNAs.

E. Violin plot showing the distribution of fold changes in *smedwi-1(RNAi)* animals relative to controls, of muscle cell transcripts encoding secreted and non-secreted proteins.

F. Violin plot showing the distribution of fold changes in *smedwi-1(RNAi)* animals relative to controls, of cathepsin cell transcripts encoding secreted and non-secreted proteins.

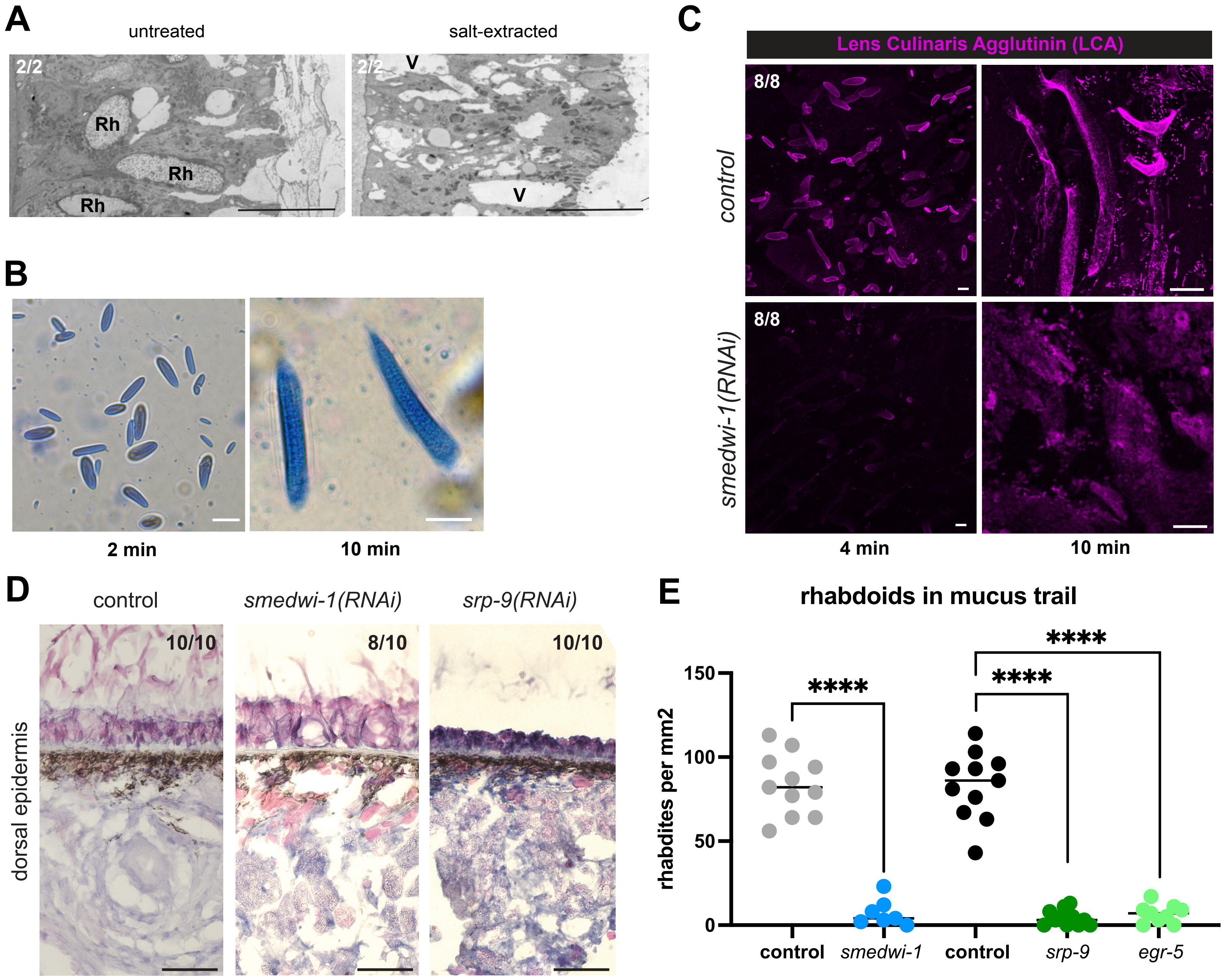

Fig. S4. (Ad Fig. 4)

Rhabdoids, or rhabdites-like structures, are rod-shaped epidermal secretory inclusions that characterize the Rhabditophora to which planarians belong, and exist in at least two types. Epidermal rhabdoids are large structures produced by cells in the epidermal layer, and upon secretion swell to produce the mucus that covers the epidermis (Hayes, 2017; Wrona, 1986). Adenal rhabdites are smaller in size and are produced from gland cells located in the subepidermal space (Smith et al., 1982). Both types of structures may be secreted upon stress and injury and may assist in physical barrier formation and microbial defense. Additional epidermal inclusions such as Hyman vesicles (Cheng et al., 2018) have been described.mi Rhabdites/rhabdoids have been primarily described based on EM imaging. Connecting the structures observed in the fixed tissues of EM images to the structures secreted from live planarians is problematic, especially as the secreted structures rapidly change shape. In EM images, the rhabdites are visible as large granules of 2-10µm in length with fibrillar or speckled content. Additionally, smaller (1-2µm) rod-shaped structures with more dense, often laminar content are present in the epidermal cells. It is possible that these smaller structures are precursors to the larger rhabdites, but it is also possible that their biogenesis is independent. Another possibility is that the large rhabdites actually represent partially swollen versions of the smaller structures.

Development • Supplementary information

Rhabdites from live animals can be observed in mucus trails or epidermal scrapes. Additionally, rhabdites are efficiently secreted upon exposure of the animals to high salt media. Immediately after secretion (in mucus trails or upon salt extraction), rod-like structures of around 10μm in length are detected, as well as some smaller rod-shaped structures. Within a few minutes, the structures swell up and rapidly turn into smears of mucus that merge with each other or form fibrous connections over long distances.

A. Electron microscopy image of salt-extracted epidermal cells shows the absence of larger rhabdoid structures (Rh) that were found in control epidermal cells, and instead shows empty spaces (V) in the epidermal tissue. This suggests that the larger rod-shaped inclusions are the primary structures that are secreted. We however cannot determine whether the smaller rod-shaped structures observed in the epidermis also contribute to the secreted objects. To avoid misnaming the structures, we therefore chose to refer to all rod-shaped secreted objects as "rhabdoids", intending this to mean rhabdite-like structures. This naming is in accordance with previous literature (e.g. (Smith *et al.*, 1982)).
The rhabdoids that were lost from the epidermis after salt extractions were between 2 and 8μm in length. Scale bar, 10μm. **B.** Aniline blue staining of the structures secreted after salt extraction. Rhabdoids of ~10μm in length were detected immediately after secretion, and rapidly increased in size. Scale bar, 10μm. **C.** Labeling of the structures secreted after salt extraction with the Rhodamine-coupled lectin LCA. Labeling shows rhabdoids of ~10μm in length immediately after secretion, that rapidly increased in size and spread out across the surface. The structure of rhabdoids from *smedwi-1(RNAi)* animals was altered and contained fewer glycoproteins that were labeled by LCA. Scale bar, 10μm.
D. (expanded from main Fig. 4a, to include the *srp-9(RNAi)* sample) Transverse sections stained with Hematoxylin and Eosin (H&E) show the altered structure of the epidermis in *smedwi-1(RNAi)* animals, including a less pronounced basement membrane (BM), and subepidermal accumulations of material (black arrowheads). Scale bar, 50μm.
E. (ad main Fig. 4d) Quantification of the secreted rhabdoids indicates a reduction of rhabdoids in *smedwi-1(RNAi)* animals, *egr-5(RNAi)* animals, and *srp-9(RNAi)* animals. Separate sets of controls were used. Datapoints represent biological replicates (n=10-12). Statistics are based on t-test. **** $p<0.0001$.

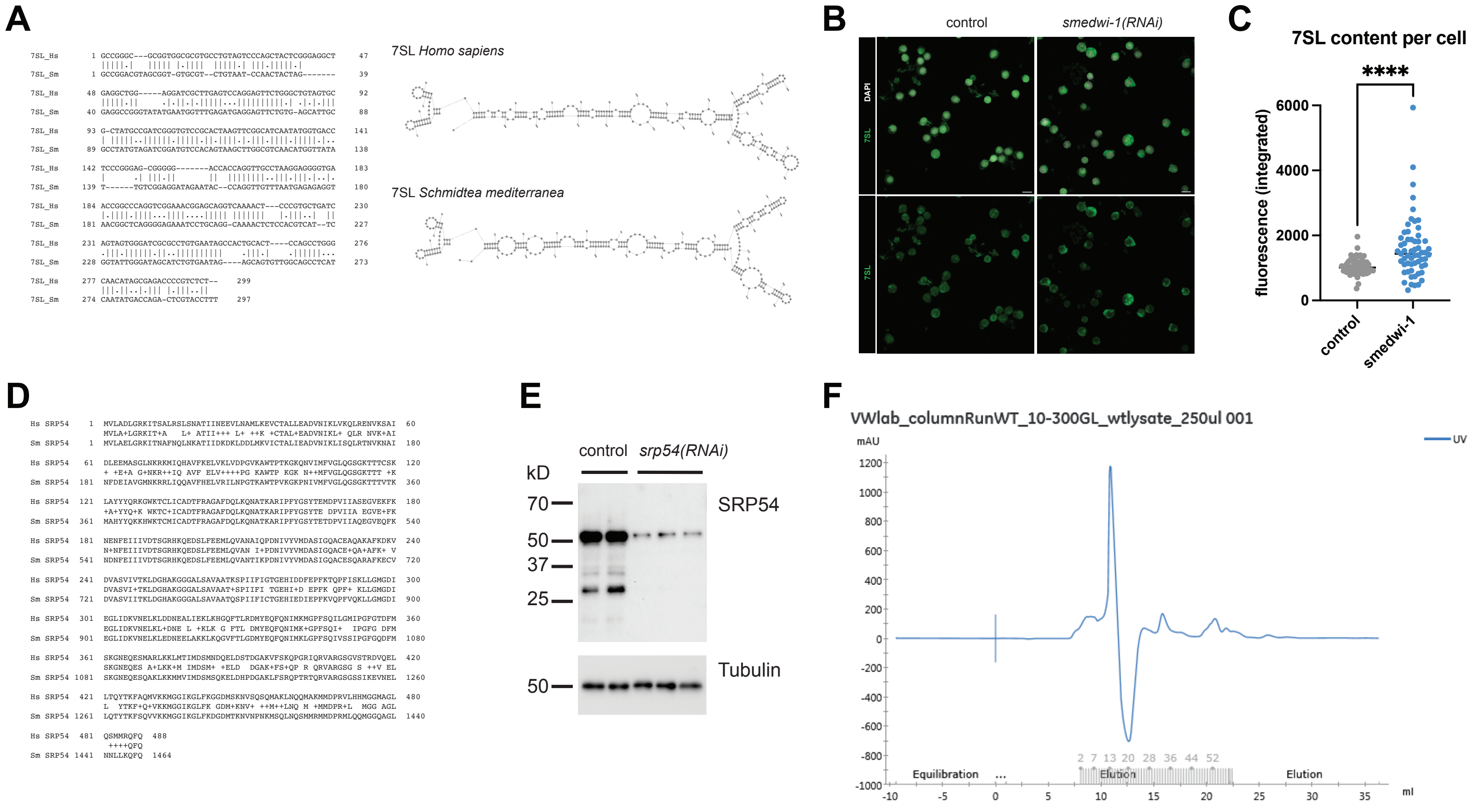

Fig. S5. (Ad Fig. 5)

A. Alignment (left) and predicted structure (right, RNAfold) of the human 7SL RNA and the predicted 7SL RNA from *Schmidtea*. The sequence diverges (62% identity), but the structure is highly conserved.

B. (expanded view from Fig. 5c) RNA FISH for the *7SL* RNA on isolated cells shows increased intensity and prominent accumulation of the RNA in the cytoplasmic space in *smedwi-1(RNAi)* samples compared to controls. Scale bar, 10µm.

C. Quantification of the 7SL signal per cell. Statistics are based on t-test. **** $p<0.0001$.

D. Alignment of protein sequence of *S. mediterranea* SRP54 to human SRP54 protein.

E. Western blot of control and *srp54(RNAi)* samples labeled with the SRP54 antibody shows a major band around 54kD in planarian lysate, that is strongly reduced upon *srp-54* knockdown, confirming antibody specificity.

F. UV trace of gel filtration column showing the full spectrum of the elution and the fractionation strategy.

Development • Supplementary information

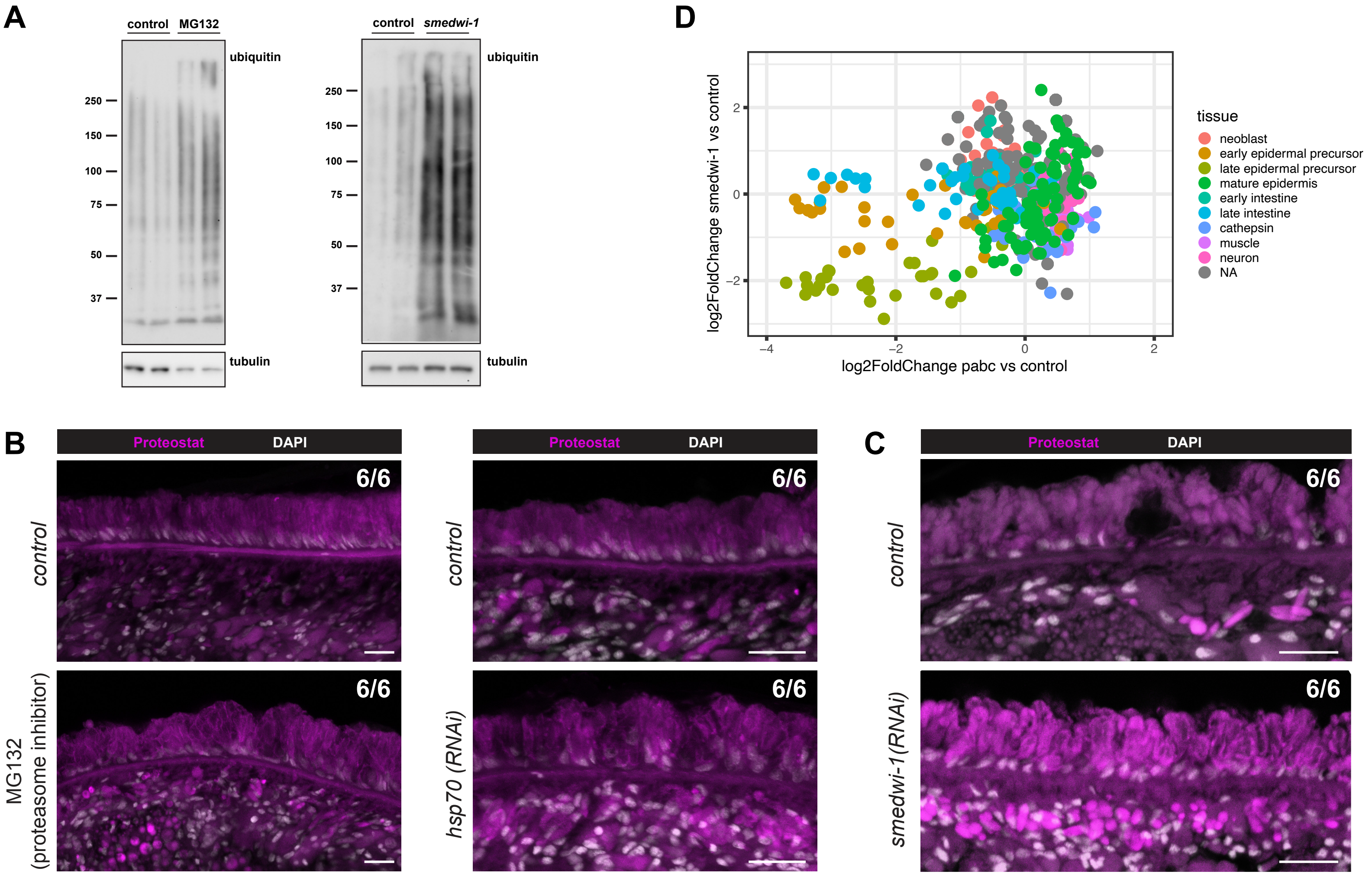

Fig. S6. (Ad Fig. 6, Discussion)

A. Western blot labeled with antibody against ubiquitin shows proteins targeted for degradation. Chemical inhibition of the proteasome by MG132 resulted in a mild increase in ubiquitinated protein, verifying the detection method. Long-term absence of SMEDWI-1 resulted in a stronger increase in ubiquitinated protein. Shown are biological replicates.

B. Transverse sections stained with the aggregation-specific protein dye Proteostat show an increase in labeling upon exposure of animals to the proteasome inhibitor MG132 or upon knockdown of the protein chaperone *hsp-70*.

C. Transverse sections stained with Proteostat show the increased presence of protein accumulations in *smedwi-1(RNAi)* animals in the epidermal cells and in the subepidermal space (arrowheads). Scale bar, 20µm.

D. Comparison of log2 FoldChanges of tissue-specific transcripts between *smedwi-1(RNAi)* samples and *pabp(RNAi)* samples (Bansal et al., 2017).

Development • Supplementary information

Table S1.

qPCR primers

	FW	RV
smedwi-1	GTCTCAGAAAACAACTAAAGGTACAGCA	TGCTGCAATACACTCGGAGACA
nanos	GGCCATGCTTCCAACAATGC	TTGCAAAGACAGATTTTTCACTGTTAGA
egr2	ACCGGTCGTCAGATGGATCC	CGCGATCTCATGAGCACAACA
egr3	ACCCCTTCTCCAATGGATTGC	TGAGACGAATCTGGAATAATTGTGGA
runt1	GTCGGCGAGTAATCCGTCGA	TATTTTCCTCTTCCACTGCGACTGA
h2b	GCATCTGAGGCAAGCAAATTGGGAGAAAGTTGA	TCAACGACGTTTTAAAAGTTCAGAACA
wntP1	GCTCAAACTGTCGCTGAGGC	CGTTGTCGTCACATCCTTGCC
wntless	TCGTCAGTCAGATTGATGAAAGTCA	TGACGTAGAAATTCCACATGCCA
prog-1	GCAATCTGCTTTCGTAATGTGTCCT	TCTGCAAAGTCTCCCGCCAA
c11136	TGCTCTTCGCAGCAATTGTGA	TGCCGAACCGCATTTGCTTT
c11136-pre	ACCGCATCCATGGATCACGT	TGCCGAACCGCATTTACTGAAAAA
c7016	TCGGCTTGTTAATTCTCAACGGC	CGGCTTCTTCTGTTACTTCAAAATTTCC
c7016-pre	CAAAGGAAATTTTGGTGTGATTTATTGAGT	CCATTTCTTATCTCCTTGAATTTCGGC
c2926	TGTGGAAACCAAGGACAATTCAGAAT	ACAAGAACCATAACAAAGTCGGAAGT
c2926-pre	CAAGGATGTGGAAACCAAGGACA	AAATCAAACATTTAACACTTTAAAGTCGCA
c6175	TGTTCTCCACGATGTGACTTGGA	TTGCCGCATATCCACCGTTG
c9505	AATGTTGAATCACGACAAACGGCT	AAGCTGACACGCACCAGTTG
vimentin	AACCGCGGCTTCAACTGAAC	CAGCGGAACTTAAAACTCGCTCTT
PRSS12	TCGAGATATTATTGGGATTCCGCA	CGGTTACGGTTGAGTATTGAGGA
c9830	TGCACTTGGTGAGGTTTTCGG	CCTTGTCCATTTTCCTCCTGGG
d558	TGGTGCTCCAATGATGAGGGA	TCTTTTGTTCTTTTTCACTTTCAGCGA
c5568	TGTTTTAATCGGTTTAGTGGCAATCGT	CCTTCAAGTCTTCGAACTCATTCAAAAG
b-gal	CTTGCGGTTCTTCTGATAGACGT	TCAGTCCACGGACCACATCA
hspA8	GAAATCGCACAACGCCCAGT	CAATCGCTTTGCATCAAAAACCGT
atg1	TCACGGTCGACCCTCCTAGA	GGCGTCAGATTTTGGTTGTGGT

cloning primers

	FW	RV
egr-5	CTGTTTCTCCCTTGATACTCCCA	ACGTATTCGGGATTTTGAACGG
srp-9	TTAGCCGATCCTTCTAAATGCAG	AATGCAAAATTATGCTCTTGAGGC
srp-54	AGCTGTTGGAATGAATAAACGTCG	TAGAGATTGTCGCGATGGCC
c6175	TGTCAAAACTTTCGATCTTTGCGT	GGAGCTTTCAGTCTCCTTGAAC
c9505	GCAAACGTCAAAGAAATGCGAC	GCCTTTGTTCCTTCCAGTGC
colf-1	TTGGTGGCTCAGGACCTCT	TTCTGACCGGACGTTCACTG
colf-2	TCTAACCGGTCCAAATGGCT	ATGGACCAAGATCTCCCGGA
colf-3	CCCGGCTTTCCTGGTAAGAA	CACCGACAACCTACTGCAGT
colf-4	TGACTATGGGAAGATCAGGTGA	TGAGGTCCAGGATCTCCAGG
colf-5	TCTTCAGGCTCACCAGGTCT	TCTTTACCGGCTGGACCAAC
colf-6	TGGAAATCCCGGTGAAGATGG	TTCCTTGTGGGCCAGGTTTT
colf-7	TGGGATTTCCTGGCATTGCA	CCTCTAGACCAGGGCTTCCT
colf-8	AGGTGATCCTGGACCTAAAGGA	CCATCAAGCCCATTGGACCT
colf-9	TGACAGCTGTACAAATTTCGGA	AATGCCTCTTGGACCCGAAG
colf-10	AGATGCATCCCATTCAATGTCT	CCCGGGTTTCCTCCAATACC
hsp70	CGCTGAACGCAATGTGTTAA	CTTGTTTTCCAGCCCCTCCT

probes

	RC
7SL	CTCTCATTAAACAACCTGGGTATTCTATCCTCCGACAATATAACCATGTTGAC
tRNA	ATGAGACTAAATCAAACATTATCTCCAAGTTAACAGCTTGGAATTTTTAATAAACTAAGTCTC

Development • Supplementary information

References

Bansal, D., Kulkarni, J., Nadahalli, K., Lakshmanan, V., Krishna, S., Sasidharan, V., Geo, J., Dilipkumar, S., Pasricha, R., Gulyani, A., et al. (2017). Cytoplasmic poly (A)-binding protein critically regulates epidermal maintenance and turnover in the planarian Schmidtea mediterranea. Development *144*, 3066-3079. 10.1242/dev.152942.

Cheng, L.C., Tu, K.C., Seidel, C.W., Robb, S.M.C., Guo, F., and Sanchez Alvarado, A. (2018). Cellular, ultrastructural and molecular analyses of epidermal cell development in the planarian Schmidtea mediterranea. Dev Biol *433*, 357-373. 10.1016/j.ydbio.2017.08.030.

Fincher, C.T., Wurtzel, O., de Hoog, T., Kravarik, K.M., and Reddien, P.W. (2018). Cell type transcriptome atlas for the planarian Schmidtea mediterranea. Science *360*. 10.1126/science.aaq1736.

Hayes, M.J. (2017). Sulphated glycosaminoglycans support an assortment of planarian rhabdite structures. Biol Open *6*, 571-581. 10.1242/bio.024554.

Smith, J., Tyler, S., Thomas, M.B., and Rieger, R.M. (1982). The Morphology of Turbellarian Rhabdites - Phylogenetic Implications. T Am Microsc Soc *101*, 209-228.

Wrona, F.J. (1986). Distribution, Abundance, and Size of Rhabdoids in Dugesia-Polychroa (Turbellaria, Tricladida). Hydrobiologia *132*, 287-293. Doi 10.1007/Bf00046262.
